# Effect of bariatric surgery on HDL-mediated cholesterol efflux capacity

**DOI:** 10.3389/fcvm.2024.1469433

**Published:** 2024-11-07

**Authors:** O. Castañer, K. A. Pérez-Vega, S. Álvarez, S. Vázquez, A. Casajoana, G. Blanchart, S. Gaixas, H. Schröder, M. D. Zomeño, I. Subirana, D. Muñoz-Aguayo, M. Fitó, D. Benaiges, A. Goday, A. Oliveras

**Affiliations:** ^1^ Cardiovascular Risk and Nutrition Research Group, Hospital del Mar Research Institute, Barcelona, Spain; ^2^ Network Biomedical Research Center Consortium (CIBER), M.P. Epidemiology and Public Health (CIBEResp), Instituto de Salud Carlos III, Madrid, Spain; ^3^ Network Biomedical Research Center Consortium (CIBER), M.P. Pathophysiology of Obesity and Nutrition (CIBERobn), Instituto de Salud Carlos III, Madrid, Spain; ^4^ Nephrology Department, Hospital del Mar, Barcelona, Spain; ^5^ Esophagogastric and Bariatric Surgery Department, General Surgery Service, Hospital del Mar, Barcelona, Spain; ^6^ Department of Nutrition, Ramon Llull University, Barcelona, Spain; ^7^ Network Biomedical Research Center Consortium (CIBER), M.P. Cardiovascular Diseases (CIBERcv), Instituto de Salud Carlos III, Madrid, Spain; ^8^ Cardiovascular Epidemiology and Genetics Research Group, IMIM, Barcelona, Spain; ^9^ Department of Endocrinology and Nutrition, Hospital del Mar, Barcelona, Spain; ^10^ Department of Medicine, Universitat Pompeu Fabra, Barcelona, Spain; ^11^ Department of Endocrinology and Nutrition, Consorci Sanitari de L'Alt Penedès I Garraf, Vilafranca del Penedès, Spain; ^12^ Department of Medicine, Autònoma de Barcelona University, Bellaterra, Spain

**Keywords:** cholesterol efflux capacity, obesity, weight loss, HDLc, lipid profile

## Abstract

**Background:**

Bariatric surgery (BS) is the most effective intervention for severe obesity, leading to sustained weight loss, reduced obesity-related comorbidities, and cardiovascular mortality.

**Aim:**

To assess changes in high-density lipoprotein (HDL) functions [cholesterol efflux capacity (CEC) and anti-inflammatory capacity] at different follow-up times in patients with severe obesity undergoing BS.

**Methods:**

A prospective observational study within a cohort of consecutively enrolled patients with severe obesity scheduled to undergo BS. In total, 62 participants (77% women), with a mean age of 42.1 years (SD 9.33 years) underwent BS. Regarding the surgical procedure, 27 (43.5%) underwent sleeve gastrectomy and 35 (56.5%) Roux-en-Y gastric bypass. All patients were evaluated preoperatively and at 1, 3, 6, and 12 months after surgery.

**Results:**

A decrease in body mass index and an improvement in the systemic lipid profile, indicated by reductions in total cholesterol, low-density lipoprotein cholesterol (LDLc), and remnant cholesterol, and an increase in HDL cholesterol (HDLc) was observed (all *p* trend < 0.001). Time-series comparisons vs. baseline showed that, in general, anthropometric measures, glycemia, total cholesterol, LDLc, and remnant cholesterol decreased at all follow-ups, whereas HDLc and triglyceride concentrations significantly improved vs. baseline from 6 months, reaching at 12 months the highest HDLc levels (29.6%, *p* < 0.001) and the lowest circulating triglycerides (−30%, *p* < 0.001). Although HDL's anti-inflammatory ability worsens after surgery, the HDL-mediated CEC linearly increased after surgery (for both *p* trend < 0.013).

**Conclusion:**

BS improves the lipid profile both quantitatively and qualitatively after 1 year, specifically enhancing HDL-mediated cholesterol efflux capacity, which may contribute to a reduced cardiovascular risk in individuals with severe obesity.

## Introduction

Obesity is a global health issue affecting over 650 million individuals worldwide. Within Spain, the prevalence of obesity stands at approximately 22% (1). Data from the World Health Organization (WHO) indicates a consistent linear rise in global obesity rates since 1975 (2). While lifestyle modifications remain the cornerstone of weight management and overall health improvement, bariatric surgery (BS) has emerged as the most effective intervention for patients with severe obesity to date and induces sustained long-term weight reduction associated with decreased obesity-associated comorbidities and cardiovascular mortality (3, 4). Indeed, the documented Swedish Obese Subjects (SOS) study revealed a significant reduction in the incidence of mortality from cardiovascular events in individuals who had undergone BS (5). The reduction in cardiovascular events could only in part be attributed to weight loss, implying a contribution from other factors. Long-term effects of BS have included an improved lipid profile, in particular, a total cholesterol decrease and a high-density lipoprotein cholesterol (HDLc) level increase (6) by 30% compared to preoperative levels and compared to individuals undergoing medical therapy alone for weight loss (7%) (6, 7). Even HDLc levels increase after surgery and remain stable regardless of weight regain after bariatric surgery (8). However, the implications of BS for the functional aspects of HDL, particularly its cholesterol efflux capacity (CEC), remain ambiguous, with conflicting findings across existing literature.

HDL particles display a wide spectrum of atheroprotective activities such as effluxing cellular cholesterol, improving endothelial dysfunction (by improving the inflammatory response and vascular constriction, and diminishing cellular death), protecting low-density lipoprotein (LDL) from oxidation, and improving glucose metabolism, among others (9). HDL plays a key role in reverse cholesterol transport which is a crucial process for the elimination of the remaining cholesterol of an organism. HDL's main ability to remove cholesterol from macrophages, known as CEC, is the first step in reverse cholesterol transport. In this regard, CEC is considered a better indicator of HDL function than circulating HDLc levels alone. An alteration in CEC may directly affect extracellular matrix composition, endothelial function, and vascular smooth muscle cell function, favoring an aortic stiffness phenotype (10), which is implicated in cardiovascular risk. HDL has the ability to reduce endothelial cell adhesion molecule expression, such as vascular cellular adhesion molecules (VCAMs), intercellular adhesion molecules (ICAMs), and e-selectin, mediated by the SR-BI and sphingosine-1-phosphate receptors (11).

Understanding how BS impacts HDL functionality could provide insights into the associated cardiovascular risk modification. Obesity leads to an abnormal metabolism of HDL, which is associated with an altered function of the HDL particles (12, 13). Studies have reported that cholesterol CEC appears to be inversely correlated with body mass index (BMI) (14). There is a decrease in the overall ability of HDL to remove cholesterol from fibroblasts in individuals with obesity (15). However, dietary weight loss in the short term did not improve ABCA1-mediated cholesterol efflux in men with abdominal obesity (14). Since CEC is the main metric of HDL function and has a strong inverse association with both sub-clinical atherosclerosis and coronary events (16–18), the reduction of CEC in obesity may have a crucial impact on the development of cardiovascular disease.

The interplay between different metabolic pathways affected by BS and their impact on CEC through HDL remains an area of ongoing investigation. Herein, we sought to assess changes in CEC at different follow-up times in patients with severe obesity undergoing BS.

## Material and methods

The BARIHTA study (bariatric surgery and arterial hypertension) was a prospective observational study in a cohort of consecutively enrolled patients with severe obesity scheduled to undergo BS (clinicaltrials.gov identifier: NCT03115502). Thus, the BARIHTA study prospectively recruited outpatients with severe obesity who went to consultations at the Hospital del Mar (Barcelona, Catalonia, Spain) seeking surgical treatment. All individuals with a medical indication for surgical intervention and who agreed to undergo BS treatment were invited to participate. Indications for BS included those patients with grade III (BMI ≥ 40 kg/m^2^) or grade II obesity (BMI ≥ 35 kg/m^2^) and associated comorbidities [i.e., type 2 diabetes mellitus (T2DM), obesity-associated hypoventilation disorders, high blood pressure, or dyslipidemia]. Patients with any endocrine disease-caused obesity or severe psychiatric diseases were excluded. Detailed information on the trial was provided by qualified professionals (19). The exclusion criteria for the BS program included an unfavorable psychiatric evaluation, a diagnosis of severe disease or any other condition that makes adherence to the BS clinical management protocol impossible, or refusal to give consent. The trial was approved by the local institutional ethics committee in accordance with the Declaration of Helsinki, and written informed consent was obtained from all participants.

The mean age of included patients was 42.1 years (SD 9.33 years) and all met the 1991 BS criteria of the National Institutes of Health (20). The indication for the type of surgical procedure, either laparoscopic sleeve gastrectomy (LSG) or laparoscopic Roux-en-Y gastric bypass (LRYGBP), was based on clinical criteria and the consensus of the BS unit.

All patients were evaluated preoperatively and at 1, 3, 6, and 12 months after surgery. Changes in anthropometric parameters, blood pressure, arterial stiffness–related markers, and indicators of renal and cardiac dysfunction have been previously published (19, 21–23).

### Laboratory analysis

Blood samples were obtained in a fasting state, processed, and stored at −80°C until further analysis. Glucose, total cholesterol, and triglycerides were determined using enzymatic methods in a Cobas Mira automatic analyzer (Baxter Diagnostics AG, Düdingen, Switzerland). HDLc was measured using separation by precipitation with phosphotungstic acid and magnesium chloride, and LDL cholesterol (LDLc) concentration was estimated using the Friedewald formula. Remnant cholesterol was calculated as total cholesterol − HDLc − LDLc.

We tested the functionality of HDL in two aspects: HDL-mediated CEC and the anti-inflammatory ability of HDL particles. The CEC analyses were performed in apolipoprotein B-depleted plasma samples (laboratory specimens enriched with HDL) from plasma aliquots stored at −80°C until use. THP-1 monocyte cells were grown in RPMI 1640 medium. We incubated the cells (52,500 cells/plate well) with phorbol-myristate-acetate (200 nM, Sigma) for 24 h to differentiate them into macrophages. For the determination of CEC, cells were incubated with fluorescent BODIPY cholesterol 0.025 mM for 24 h, washed, and incubated for a further 24 h in fresh serum-free RPMI 1640 medium. After washing the cells, we incubated them with 6.6% apolipoprotein B-depleted plasma from the volunteers for 16 h. We then collected the supernatants, incubated the cells with 1% Triton X-100 for 60 min, and determined the fluorescence in both specimens (485/535 Ex/Em) in an Infinite M200 reader (Tecan Ltd.). After subtracting blank fluorescence from the other measurements, we calculated the cholesterol efflux capacity values as follows: [fluorescence in supernatants/(fluorescence in supernatants + fluorescence in cells)] × 100. Finally, we obtained the adjusted cholesterol efflux capacity values by subtracting the efflux in the negative controls from the efflux values of the samples (18). Finally, the anti-inflammatory ability of HDL was assessed by measuring the secretion of VCAM after a pro-inflammatory stimulus with tumor necrosis factor (TNF) molecules in the human umbilical endothelial vascular cells (HUVEC) model. HUVEC cells were cultured in endothelial growth medium-2 (EGM-2) at a density of 47,500 cells per well. After washing, the cells were incubated with a 10% dilution of apolipoprotein B-depleted plasma in EGM-2 medium. Following another wash, TNF-alpha (10 ng/ml per well) was added, and the plates were incubated in a CO_2_ environment for 24 h. After incubation, the supernatant was collected for subsequent measurement of soluble VCAM using ELISA methodology (R&D Systems, Minnesota, USA) (24). The anti-inflammatory HDL ability is inversely related to the VCAM secretion quantification.

### Surgical techniques

The LRYGBP technique consisted of a 150-cm antecolic Roux limb with a 25-mm circular pouch-jejunostomy and a 50-cm exclusion of the proximal jejunum. In sleeve gastrectomy, a longitudinal resection of the stomach from the angle of His to approximately 5 cm proximal to the pylorus was performed using a 36 Fr bougie inserted along the lesser curvature. The same team of surgeons performed all operations.

### Statistical analysis

Data were expressed as mean ± standard deviation for continuous variables following a normal distribution. The rest of the continuous variables were log-transformed to achieve normality. Normality was checked visually and using the Kolmogorov–Smirnov test. Student's *t*-test was performed to assess the differences between two means. Chi-square or Fisher's exact tests were used to evaluate the degree of association among categorical variables. Linear mixed models for repeated measurements adjusted for age, sex, type of intervention, and treatment of dyslipidemia were fitted to study the evolution of the continuous variables in each group and analyze differences between groups at each time point from baseline. The coefficient of change per month was calculated, as well as the *p*-values for the linear trend effect. We modeled the trajectories of risk factors using smoothed cubic spline mixed effects regression models.

A two-sided *p*-value <0.05 was considered statistically significant. Statistical analysis was calculated with R (version 4.3.3) (25).

## Results

In total, 62 participants (77% women) from the Obesity Unit in Hospital del Mar underwent BS. The mean age of included patients was 42.1 years (SD 9.33 years), with an age range for women and men of 32–53 and 21–60 years, respectively. Regarding the surgical procedure, 27 (43.5%) underwent LSG and 35 (56.5%) LRYGBP. Anthropometric parameters and glucose decreased linearly after surgery during the 12 months of follow-up (*p* trend < 0.001). Furthermore, reductions in total cholesterol, LDLc, and remnant cholesterol, and an increase in HDLc (all *p* trend < 0.001) were observed (Table 1).

**Table 1:** General characteristics of the participants, lipid profile, and CEC promoted by HDL, over time.

*N* = 62	Baseline	Follow-up post bariatric surgery				Coefficient	*p* trend
		1 month	3 months	6 months	12 months		
Weight (kg)	117 (18.9)	105 (17.0)*	95.0 (16.0)*	86.6 (16.8)*	82.0 (16.5)*	−0.13 (−0.14, −0.11)	<0.001
BMI (kg/m^2^)	42.6 (5.49)	38.0 (5.05)*	34.5 (4.71)*	31.3 (5.05)*	29.7 (4.82)*	−0.14 (−0.16, −0.13)	<0.001
Abdominal circumference (cm)	132 (11.5)	122 (10.2)*	116 (10.1)*	110 (13.3)*	105 (13.3)*	−0.14 (0.15, −0.12)	<0.001
Glucose (mg/dl)	101 (21.0)	90.8 (15.7)	87.2 (6.59)*	88.2 (7.39)*	89.3 (9.24)*	−0.04 (−0.06, −0.02)	<0.001
Total cholesterol (mg/dl)	176 (28.3)	147 (41.7)*	162 (36.1)*	162 (33.5)*	161 (27.0)*	−0.01 (−0.03, 0.01)	0.174
LDL cholesterol (mg/dl)	115 (25.0)	95.9 (33.6)*	104 (28.9)	99.8 (28.1)*	95.0 (24.3)*	−0.04 (−0.06, −0.02)	<0.001
HDL cholesterol (mg/dl)	46.9 (12.9)	38.9 (11.0)*	45.6 (11.8)*	51.7 (11.8)*	60.8 (14.2)*	0.10 (0.09, 0.12)	<0.001
Remnant cholesterol (mg/dl)	14.0 (7.53)	16.4 (7.17)*	14.2 (8.20)	10.5 (5.87)*	7.16 (5.67)*	−0.09 (−0.12, −0.07)	<0.001
Triglycerides (mg/dl)	105 (46.8)	98.4 (39.2)	90.9 (38.3)	79.1 (27.7)*	73.5 (30.1)*	−0.07 (−0.09, −0.06)	<0.001
HDL Cholesterol efflux capacity (%)	0.98 (0.15)	0.97 (0.14)**	1.03 (0.11)	1.00 (0.14)	1.04 (0.13)**	0.04 (0.01, 0.07)	0.010
HDL VCAM secretion (ng/ml)	6.83 (2.13)	7.02 (1.87)	6.71 (2.18)	7.14 (2.05)	7.61 (2.39)	0.03 (0.01, 0.03)	0.013

BMI, body mass index; LDL, low-density lipoprotein; HDL, high-density lipoprotein.

Baseline and follow-up values are presented as mean (SD). Model adjusted by age, sex, type of intervention, and treatment of dyslipidemia.

* *p*-value < 0.05.

** Borderline *p*-value < 0.01.

When time-serial vs. baseline was analyzed, glycemia improved from 3 months onward. We observed a significant improvement in anthropometric measures, total cholesterol, LDLc, and remnant cholesterol at all the follow-ups (except LDLc and remnant cholesterol at 3 months). Triclyceride levels showed a decrease at 6 and 12 months. With regard to HDLc, a decrease was observed at 1 and 3 months, whereas an increase was observed at 6 and 12 months vs. baseline (Table 1).

**Figure 1:**
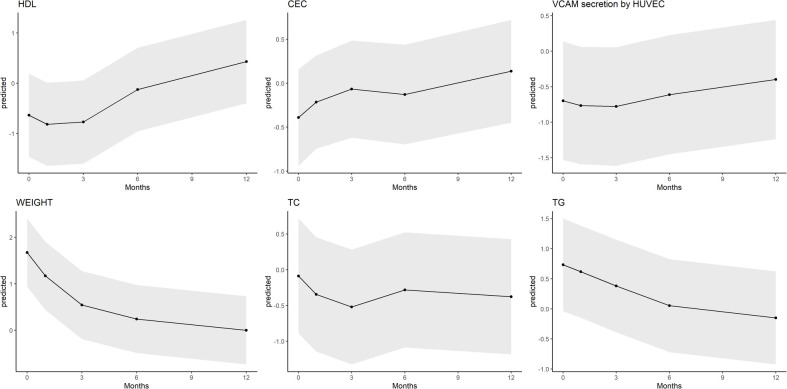
Evolution of weight, lipid profile, and HDL function-related biomarkers. HDLc, high-density lipoprotein cholesterol; CEC, cholesterol efflux capacity of HDL; AIC, anti-inflammatory capacity of HDL; TC, total cholesterol; TG, triglycerides.

CEC increased linearly for 12 months after surgery (*p* trend = 0.010), reaching the highest value at 12 months compared to baseline although the statistical significance was not reached (*p* = 0.098). However, the VCAM secretion values showed a general increase over time, suggesting a gradual decline in anti-inflammatory activity throughout the 12-month period, with a brief improvement at 3 months (Table 1). The predicted trajectories of weight, lipid profile, and HDL function are depicted in Figure 1.

We then analyzed the relationship between HDL CEC and other variables. There were no differences among the tertiles of CEC with regard to the anthropometric parameters or other characteristics at baseline nor the baseline lipid and glycemic profile of the participants (Table 2).

**Table 2:** Relationship between tertiles of HDL-mediated efflux capacity and the baseline characteristics of the participants.

	Tertiles of CEC			*p* trend
	(0.610, 0.929)	(0.929, 1.046)	(1.046, 1.292)	
	*N* = 20	*N* = 20	*N* = 19	
Age (years)	41.6 (8.93)	39.8 (8.45)	44.1 (10.6)	0.419
Sex				0.302
Men	6 (30.0%)	5 (25.0%)	3 (15.8%)	
Women	14 (70.0%)	15 (75.0%)	16 (84.2%)	
Type of BS				0.433
LRYGBP	12 (60.0%)	11 (55.0%)	9 (47.4%)	
LSG	8 (40.0%)	9 (45.0%)	10 (52.6%)	
Smoke				0.522
Yes	6 (30.0%)	6 (30.0%)	5 (26.3%)	
No	10 (50.0%)	9 (45.0%)	8 (42.1%)	
Former smoker	4 (20.0%)	5 (25.0%)	6 (31.6%)	
T2DM	4 (20.0%)	1 (5.00%)	2 (10.5%)	0.356
Hypertension	7 (35.0%)	7 (35.0%)	8 (42.1%)	0.652
Dyslipidemia	2 (10.0%)	1 (5.00%)	4 (21.1%)	0.298
Abdominal perimeter (cm)	134 (14.6)	133 (10.9)	131 (9.09)	0.450
BMI (kg/m^2^)	41.9 (5.25)	44.6 (5.71)	41.6 (5.49)	0.885
Log-glucose (mg/dl)	4.63 (0.24)	4.58 (0.18)	4.60 (0.09)	0.521
Total cholesterol (mg/dl)	169 (31.4)	177 (30.0)	181 (23.9)	0.183
LDL cholesterol (mg/dl)	111 (27.6)	116 (25.5)	118 (23.2)	0.402
HDL cholesterol (mg/dl)	44.6 (14.5)	47.5 (14.8)	48.7 (9.60)	0.340
Remnant cholesterol (mg/dl)	12.6 (7.54)	14.6 (8.51)	14.0 (6.72)	0.561
Log-triglycerides (mg/dl)	4.47 (0.35)	4.63 (0.51)	4.59 (0.39)	0.362
Anti-inflammatory HDL ability (ng/ml)	5.92 (2.37)	7.11 (2.17)	7.21 (1.70)	0.077

BS, bariatric surgery; BMI, body mass index; LDL, low-density lipoprotein; HDL, high-density lipoprotein; LRYGBP, laparoscopic Roux-en-Y gastric bypass; LSG, laparoscopic sleeve gastrectomy; T2DM, type 2 diabetes mellitus.

Baseline and follow-up values are presented as mean (SD).

## Discussion

BS is currently considered the most effective intervention for patients with severe obesity, especially in terms of sustained long-term weight reduction associated with decreased obesity-associated comorbidities and cardiovascular mortality (26, 27). Effective remission of hyperlipidemia in patients with morbid obesity has been observed, with most patients no longer requiring lipid-lowering agents within 6 months after surgery (28). We document here that BS produced favorable metabolic changes in individuals with obesity who lost weight after the intervention, such as the classical lipid profile which behaved positively, as expected. Furthermore, in this study, we reported the effect of BS on HDL functionalities, measured by *in vitro* CEC and the anti-inflammatory abilities promoted by HDL particles in the two cell types specifically involved in atherogenesis, namely, macrophages and endothelial cells. Beyond an increase in the HDLc concentration, we observed a significant improvement in CEC (following a linear trend from 0 to 12 months) with the highest increase occurring 1 year after BS. These results are in concordance with the literature. BS is associated with an improvement in CEC, a decrease in BMI, an improvement of the systemic lipid profile, and a decrease in total cholesterol, LDLc, and remnants of cholesterol.

Other studies have assessed HDL functionalities with mixed results that varied depending on the surgical procedure, the methodology to assess CEC, and the studied population. Within the STAMPEDE study designed to study a T2DM population after BS compared with medical treatment, CEC improved at 6 months after LSG, while it took 1 year after LRYGBP (29). Only 5 years after surgery did CEC increase significantly in both surgical groups, compared to the medical therapy group (29). Another study that assessed the effect of LSG in young Hispanic women who were premenopausal demonstrated an increase in CEC after 1 year (30). The observed positive results in CEC described in the literature, especially in LRYGBP, suggest that the metabolic pathways by which HDL improves its CEC function could be different depending on the surgery. In the present study, we analyzed BS as a whole, adjusting by type of intervention but not separately because of the resultant loss of statistical power.

Human trials suggest that within the first 6 months during rapid weight loss with BS, both HDLc and Apo E decrease. The initial drop in HDL cholesterol levels may reflect the gradual qualitative switch in HDL particles from Apo E-containing to more functional Apo A1-containing HDL particles, which may explain the improvement in HDL structure and functionality (31). Indeed, we expected that HDL particles would exhibit both an enhanced anti-inflammatory potential and improved cholesterol efflux. However, soluble vascular cellular adhesion molecule (sVCAM) secretion levels showed a general increase over the examined period. Therefore, further cell-based experiments using a broader panel of inflammation-related biomarkers are necessary to determine the potential recovery of HDL's anti-inflammatory action. Compared to lean adolescents, an adolescent population with obesity, some with combined T2DM diabetes, exhibited an altered HDL subspecies profile with enrichment in small HDL particles (32). These early changes in the lipid and protein compositions of different HDL subspecies in adolescents with type 2 diabetes are related to early markers of arterial disease. In this regard, analyzing the composition of HDL, rather than HDLc, may be useful in assessing cardiovascular risk in this population (32). Furthermore, mean CEC has been reported to be 12% higher in participants post BS, with an improvement in other HDL functions such as HDL lipid peroxidation and HDL anti-oxidative capacity (33) or a more cardioprotective HDL subfraction profile (34). Thus, on the whole, our findings agree with other studies that report a better HDL function as measured by CEC.

Besides the ability of HDL to promote CEC, there is increasing evidence that HDL-mediated antiatherogenic actions toward the endothelium function have physiological relevance (9). Various functions of HDL have been evaluated in several studies together with CEC, such as oxidation and vasodilation. For instance, a study involving 20 individuals with severe obesity (BMI > 50 kg/m^2^) reported an increase in antioxidant potential, accompanied by elevated paraoxonase 1 protein levels 6 months after BS (31). Furthermore, in the adolescent population in the aforementioned study, adolescents with obesity also exhibited increased arterial stiffness when measured non-invasively by pulse wave velocity compared to controls (32). Another study assessed whether LRYGBP restores the protective properties of HDL and reverses obesity-induced endothelial dysfunction. The authors found that in both human samples and a rat model of LRYGBP, the endothelium-protective activities of HDL were enhanced. HDL isolated from patients post-LRYGBP showed restored endothelial nitric oxide synthase activity, increased nitric oxide release, reduced endothelial nicotinamide adenine dinucleotide phosphate oxidase, and decreased endothelial apoptosis and VCAM expression (35). Although we also analyzed the *in vitro* anti-inflammatory ability of HDL, the results were not conclusive for an association. Given that a patient’s state before the BS could be a favorable one due to the preparative intervention, the anti-inflammatory ability might take longer to be restored in comparison to the baseline. In a meta-analysis of 48 prospective non-randomized studies, the authors reported that BS decreases the low-grade inflammation associated with obesity as measured by C-reactive protein (CRP) and interleukin 6, but not by tumor necrosis factor α, after pooling groups at 6 and 12 months follow-up (36). Although there is an improvement in low-grade inflammation after BS, the enhancement of the vascular protective properties of HDL particles may not parallel the improvements in CEC. In a previous study using the same population as in the present work, we observed a significant reduction in circulating high-sensitivity CRP levels over the follow-up period. Therefore, we hypothesize that this improvement in systemic inflammation may have positively influenced the changes in cholesterol efflux observed.

This study has strengths and limitations. For example, most of the participants were women. Sex disparity in favor of women has been documented in other studies assessing BS effects. The analysis of HDL CEC involved patients who underwent both surgical methods collectively, rather than the analysis of an individual surgical method. Due to the limited sample size, it was challenging to distinguish the specific effects of each type of surgery on HDL function. In addition, a broader panel of inflammatory markers would provide further insight into the effects of HDL function in terms of its anti-inflammatory actions. Although we analyzed HDL function at baseline and at four follow-ups, we cannot extrapolate the results for a longer time.

## Conclusion

Lipid profile and HDL functionality, as measured by the cholesterol efflux capacity, were consistently impaired in individuals with obesity and ameliorated 12 months after bariatric surgery in a linear manner.

## Data Availability

The raw data supporting the conclusions of this article will be made available by the authors upon request, without undue reservation.
